# The prevalence of chiropractic-related terminology on South African chiropractors’ webpages: a cross-sectional study

**DOI:** 10.1186/s12998-023-00483-3

**Published:** 2023-04-03

**Authors:** F. Ismail, M. Pretorius, C. Peterson, C. Yelverton

**Affiliations:** Department of Chiropractic, Faculty of Health Sciences, University of Johannesburg, Beit Street, Doornfontein, South Africa

**Keywords:** Chiropractic, Interprofessional communication, Patient centered care, Communication, Evidence based practice, Manipulation

## Abstract

**Background:**

Effective communication is imperative for successful interprofessional collaborative interactions that augment both patient-centred and evidence based care. Inquiry into the prevalence of chiropractic-related terminology on South African chiropractor’s webpages has not been explored to date. The implications of such analysis could indicate the professions’ ability to effectively communicate in interdisciplinary settings.

**Method:**

From 1 to 15 June 2020, Google search was used to identify the webpages (excluding social media accounts) of South African private practice chiropractors registered with the Allied Health Professions Council of South Africa (AHPCSA). Webpages were word-searched for eight chiropractic terms with context: subluxation; manipulate(-ion); adjust(-ing/-ment); holism(-tic); alignment; vital(-ism/-istic); wellness; and innate intelligence. Data collected was transferred to an Excel spreadsheet. Accuracy of information was verified by the researchers through a process of double checking. The number of instances each term was used, and certain socio-demographic data were recorded. Descriptive statistics and bivariate analyses were used to summarise and analyse the data.

**Results:**

Among 884 AHPCSA-registered South African chiropractors, 336 webpages were identified and analysed. From 1 to 15 June 2020, the most commonly found terms on 336 South African chiropractic webpages were 'adjust(-ing/-ment)', 'manipulate/manipulation', and 'wellness', with prevalence estimates of 64.1% (95% confidence interval [CI], 59.0% to 69.2%), 51.8% (95% CI, 46.5% to 57.1%), and 33.0% (95% CI, 28.2% to 38.2%), respectively. The least commonly found terms were 'innate intelligence' and 'vital(-ism/-istic)', with prevalence estimates of 0.60% (95% CI, 0.16% to 2.1%) and 0.30% (95% CI, 0.05% to 1.7%), respectively. Manipulate(-ion) was used more by male chiropractors (*p* = 0.015). The longer a chiropractor was in practice the more likely they were to use profession-specific terms (*p* = 0.025). The most frequently occurring combination of terms were adjust(-ing/-ment) and manipulate(-ion), found in 38 out of 336 webpages (11.3%; 95% CI, 8.4% to 15.1%).

**Conclusion:**

The use of chiropractic-related terminology on South African chiropractic webpages was common, with the prevalence of term use varying by type of terms, by gender of the chiropractor, and by clinical practice experience. Better understanding of the effects of chiropractic terminology use on interprofessional and patient interactions and communication is warranted.

**Supplementary Information:**

The online version contains supplementary material available at 10.1186/s12998-023-00483-3.

## Background

Patient or person-centred care (PCC) is an ethical approach [1] that aims to provide healthcare that is both receptive and accountable to the patient, as it conforms to the patient’s individualised preferences and principles [2–4]. Evidence based practice (EBP), another strongly encouraged approach to healthcare, is the use of the most current evidence from systematic research coupled with practitioner clinical expertise and patient values when providing health care [5–7]. These approaches are deemed the benchmark for quality care by healthcare institutions, healthcare policy makers, practitioners, and researchers alike [5, 8–10], and are strongly dependent on the interpersonal relationships not only between the patient and the professionals providing care, but also through collaboration inter-professionally [11, 12]. These interprofessional collaborative relationships are relied upon as medical conditions become more complex [13] with a need for a diverse knowledge base that cannot be provided by a single profession alone and are necessary [13, 14] for the delivery of high-quality evidence-based patient centered care. Existing literature highlights interprofessional communication, defined as the manner in which a health professional effectively converses with colleagues, patients and families in the context of the patient’s culture, as a unanimous key competency for useful collaboration between professionals [15–18].

Often the individual members of a medical interprofessional collaborative team emerge from differing education routes, have specialized skills that use different professional languages that align them to diverse aspects of healthcare [19, 20]. Following from this there may be disparities in the understanding and interpretation of the terminologies used to communicate patient conditions and treatment options generating interprofessional communication and coordination strains [21, 22]. An additional facet to interprofessional communication for effective interprofessional collaboration is whether the terminology and language actually used by one professional is in fact understandable to other health-care professionals in order to provide accurate information to the specific health-care case [23, 24]. These concerns are more pronounced in the South African context where there is an estrangement between mainstream medical healthcare and complimentary chiropractic care [25, 26] that may be aggravated by a communication divide.

Chiropractors in South Africa have been shown to have poor interprofessional-referral relationships with other musculoskeletal professionals, with 60.2% of chiropractors almost never receiving a referral from an orthopaedic specialist [27]. However, with almost a third of South African chiropractors situated in multidisciplinary health-care offices [27] there is an imperative to encourage and sustain chiropractic-medical interprofessional collaboration through communication competency [28]. One way in which this has been supported was with the adoption of the education collaboration between international chiropractic programmes (of which both South African institutions are signatories), to detach from chiropractic-related terminologies and adopt a solely evidence-based chiropractic education. This stance would allow for effective communication in a language clearly understood by all stakeholders in health-care teams [29].

Similar international studies undertaken in Australia and the United Kingdom (UK) indicate that majority of the chiropractor websites screened in those countries included more than one chiropractic-related term. Interestingly, the statistical comparison between these two studies showed that while the chiropractic terms were used in the same order of preference (adjust (-ing/-ment) mostly used followed by wellness, holism (-tic), subluxation and finally innate intelligence) the frequency of usage was to a lesser extent in the UK [28, 30].

There is currently no literature on the prevalence of chiropractic-related words used on South African chiropractors’ websites or webpages, and whether the educational collaborative statement has had any impact on the use of chiropractic-related terminology post 2014. These insights are relevant in documenting the trends of South African chiropractor communication skills. Chiropractic-centric terminology primarily includes the words subluxation, manipulate (-ion), adjust (-ing/-ment), holism (-tic), alignment, vital (-ism/-istic), wellness, and innate intelligence, detailed in Table 1. These terms are further discussed and referenced in Additional Files 1 and 2. Therefore, the objective of this paper was to record the prevalence of these eight identified chiropractic-related words on South African chiropractors’ webpages that were used in a chiropractic-centric manner.

**Table 1:** The nine chiropractic-related terms searched for and their definitions [32–42]

Subluxation	A chiropractic concept used to describe the misalignment of a vertebra thereby applying pressure to surrounding nerves which cause eventual disease [32]
Adjust(-ing/-ment) and manipulate(-ion)	Chiropractic adjustment has been defined as a form of a manual technique administered to a joint of the spine or any other body part, through which a controlled force is used or a low amplitude, high velocity manual thrust to a motion segment or joint [33]
Holism(-tic)	Holism is the philosophy which states that many parts of a whole are so interrelated that they cannot exist individualistically [34]. An understanding of holism within health care is concerned with complete systems rather than with the analysis of, treatment of, or dissection into parts. Holism (-tic) is also not an exclusive chiropractic-related term and is an approach that involves the physical, mental and spiritual well-being of a patient [35, 36]
Alignment	The historical chiropractic rationale was to visualise the chiropractic subluxation, defined at the time as a ‘bone out of place’, irritating a nerve, and as a result causing disease. This model, often using radiography for the so-called diagnosis, has survived in some parts of the chiropractic profession, despite it lacking evidence or clinical validity [28]
Vital(-ism/-istic)	Vitalism refers to the theory that all living organisms are sustained by a vital life force that is both separate from and greater than normal physical or chemical forces. There are many ways of expressing this life force, including Qi, energy, yin-yang, universal intelligence, or innate intelligence [37]. Vitalism within the chiropractic context can be broken down into two sections: universal and individual and is the idea that a vertebra out of place (i.e. the chiropractic ‘subluxation’) is interrupting the flow of vital energy (innate intelligence) within the body and is the cause of disease or pain in the body [38, 39]
Innate Intelligence	Innate intelligence represents the force that restores health and energy within the body [38, 39] in the chiropractic vitalist background. The vitalist paradigm is an unscientific ideology that is unrecognized by other mainstream medical health professions [40]
Wellness	Wellness-based chiropractic, a form of vitalism, proposes an all-inclusive scope of practice and believes in the ability to address all features of an individual’s health that go beyond the bodily symptomatology and include passionate, mystical and mental aspects of life [41]. Wellness, in this sense, is an ideology that does not align with the mainstream public health definition of wellness that describes healthy life choices for improved mental and physical outcomes [30, 42]

## Methods

The Allied Health Professions Council of South Africa (AHPCSA) documented the registration of *n* = 884 chiropractors in South Africa at the time of the study [31]. Inclusion criteria specified only private practice formal webpages owned by AHPCSA registered chiropractors with the purpose of providing public information and education. Social media accounts were excluded. To access and appraise the available webpages the Google search engine (Google LLC, Mountain View, CA, USA) was used over a two week period from 1st June 2020 to 15th June 2020, by using the phrase “chiropractor” + [name of Province] such as “chiropractor + Gauteng”. Every webpage linked to the key words ‘chiropractor’ and individual South African provinces (‘Gauteng’, ‘KwaZulu-Natal’, ‘Free State’, ‘Limpopo’, ‘North West’, ‘Mpumalanga’, ‘Western Cape’, ‘Northern Cape’ and ‘Eastern Cape’) was screened for eligibility. The Google search engine allows for selection bias as webpages that are not skilfully marketed may not be promoted during searches, also some chiropractors may not have a webpage. However, this study intended to use information that is available to the general public and thus it was necessary to use the findings yielded by general search without any manipulation of search strategies. The methods utilised in this study and the selected chiropractic-related terms were based on an original study that investigated the prevalence of chiropractic-specific terminology on Australian chiropractors' websites by Young in 2020 [28]. Each eligible webpage appearing on the Google search was then word-searched, using the ‘command + F’ keyboard search function for eight specific words (subluxation, manipulate (-ion), adjust (-ing/-ment), holism (-tic), alignment, vital (-ism/-istic), wellness, and innate intelligence).

The use of chiropractic-related terminologies was documented based on the context and value used. Thus, any sentence that devalued the meaning of a chiropractic-related word by rejecting its chiropractic-related meaning, whilst still using the word in the sentence, was not tallied. A conjectural example of this is: “We treat vertebral subluxations” was included, but “We believe the vertebral subluxation model is only a historical model and has no clinical value” was excluded [28]. Further information such as the practitioners’ gender, university of study and the number of years in practice, if available, was recorded. In the event where a webpage housed more than one chiropractor, demographic data was only captured for the primary chiropractor determined as the practice owner. Where these demographic data were not available, a zero was placed under that website for that variable. The number of webpages screened were not proportionate to the number of practicing chiropractors in each province. The chiropractor webpage URL and relevant data was captured on a Microsoft Excel (Microsoft Corporation, Redmond, WA, USA) spreadsheet. Each record was checked for correctness by two of the three researchers. Ethical clearance and consent to participate is not applicable as this research study did not involve humans or animals thus a waiver of ethical clearance was applied for and received. Justification for this waiver application was that this study relied solely on information in the public domain.

Number of citations per page was converted into a binary categorical variable where 0 was no citation and 1 was at least one citation. Additional variables were computed, including a variable showing how many different terms occurred on a webpage and a variable that showed the combination of terms used on a webpage. The frequency procedure was used to establish how many webpages featured each term, to show the distribution of number of citations for each term, to show how many different terms occurred on a webpage, and the main combinations of terms on the webpages. The findings were reported using descriptive statistics such as frequencies with percentages, means with standard deviations where appropriate. The 95% confidence intervals for proportions was calculated using the Wilson method to report on where the population parameter was most likely located between. Bivariate analysis in the form of Pearson's correlation coefficient and chi-square test was used to assess for relationships between chiropractic terms and socio-demographic variables.

## Results

Of the 884 chiropractors in the country, 336 webpages were found through census sampling where all the webpages found were then analysed for relevant terms. Fourteen webpages were excluded as 3 of the chiropractor’s webpages were in fact not located in South Africa, 5 webpages were unable to load, and 6 webpages only mentioned the chiropractor’s name with a link to their social media accounts. A *p* value of less than 0.05 was be considered significant. IBM SPSS version 28 was used for the analysis.

### Sociodemographic characteristics of the chiropractors

A total sample of *n* = 336 webpages were appraised in this study. Gender was recorded for 288 chiropractors, where *n* = 169 (58.7%) were male and *n* = 119 (41.3%) were female. Just over 90 percent of chiropractors were located in three provinces: Gauteng (*n* = 101; 30.1%); KwaZulu-Natal (KZN) (*n* = 101; 30.1%); and Western Cape (WC) (*n* = 101; 30.1%). The remaining chiropractors were located in Eastern Cape (*n* = 17; 5.1%), Free State (*n* = 4; 1.2%), North West (*n* = 4; 1.2%), Limpopo (*n* = 3; 0.9%), Mpumulanga (*n* = 3; 0.9%) and Northern Cape (*n* = 2; 0.6%) provinces. It was possible to record number of years the chiropractor was in practice for ninety-eight chiropractors. The minimum and maximum number of years in practice were one year and 47 years respectively. The average number of years in practice was 13.83 (SD = 8.97). Place of study could be identified from 248 of the 336 webpages. Most chiropractors had obtained their qualification from a South African institution, either the University of Johannesburg (UJ) (*n* = 113; 45.6%) or the Durban Institute of Technology (DUT) (*n* = 111; 44.6%). Smaller numbers had studied at an institution either in the United States of America (USA) (*n* = 21; 8.5%) or in the United Kingdom (UK) (*n* = 3; 1.2%).

### Use of terms per webpage

Analysis of use of terms per webpage is presented in Table 2 below. Three measures were used for analysis of use of terms: number (and percentage) of webpages citing the term at least once; sum of number of times a term is used; and mean of number of times a term is used. Based on the number of webpages that cited a term, adjust(-ing/-ment), manipulate(-ion) and wellness were the three most prevalent words while subluxation, innate intelligence and vital(-ism/-istic) were the three least prevalent words (Table 2). Adjust(-ing/-ment), manipulate(-ion) and wellness were cited in 64.3%, 51.8% and 34.2% of webpages respectively. Terms innate intelligence and vital(-ism/-istic) occured in just two and one webpages respectively.

**Table 2:** Occurrence and prevalence of chiropractic-related terms among 336 South African chiropractic webpages

Term	Number of webpages that cited a term at least once (n)	Percent of webpages that cited a term at least once (%)	95% confidence interval (Score (Wilson) method)		Ranking of term based on percent of webpages that cited a term at least once	Sum of number of times a term was used (n)	Ranking of term based on sum	Mean of number of times a term was used	Ranking of term based on mean
			Lower CL	Upper CL					
Adjust(-ing/-ment),	216	64.3	59.0	69.2	1	1106	1	3.29	1
Manipulate(-ion)	174	51.8	46.5	57.1	2	611	2	1.82	2
Wellness	111	34.2	28.2	34.9	3	324	3	0.96	3
Alignment	100	29.8	25.1	34.9	4	220	5	0.65	5
Holism (-tic)	95	28.3	23.7	33.3	5	139	6	0.41	6
Subluxation	59	17.6	13.9	22.0	6	231	4	0.69	4
Innate Intelligence	2	0.6	0.2	2.1	7	2	7	0.01	7
Vital (-ism/-istic)	1	0.3	0.1	1.7	8	1	8	0.00	8

From the sum of number of times a term was used, adjust(-ing/-ment), manipulate(-ion) and wellness were also the most frequently used terms. Adjust(-ing/-ment), was used 1106 times, manipulate(-ion) 611 times and wellness 324 times. Innate intelligence and vital(-ism/-istic) were used twice and once respectively. Subluxation, which occurred in just 17.6% of webpages (ranked 6) was the fourth most frequently used term (used 231 times). Analysis of mean number of times a term was used follows a similar pattern to the sum of use. Adjust(-ing/-ment), manipulate(-ion) and wellness were the three top ranked terms based on average use (3.29, 1.82 and 0.96 citations per webpage on average). Subluxation was the fourth most used term based on mean (0.69 citations per webpage on average) and innate intelligence and vital (-ism/-istic) were used the least (Table 2). Table 3 below represents a detailed account of the eight chiropractic-related terms per province.

**Table 3:** Occurrence of eight chiropractic-related terms by South African provinces

	Province	Subluxation n (%)	Adjust(-ing/-ment) n (%)	Holism(-tic) n (%)	Alignment n (%)	Vital(-ism/-istic) n (%)	Wellness n (%)	Manipulate(-ion) n (%)	Innate intelligence n (%)	Total n (%)	95% confidence interval (Score (Wilson) method)	
											Lower CL	Upper CL
	GP	47 (20.4)	381 (34.5)	21 (15.1)	78 (35.5)	0 (0.0)	57 (17.6)	180 (29.5)	2 (100.0)	766 (29.1)	27.4	30.8
	KZN	61 (26.4)	234 (21.2)	45 (32.4)	42 (19.1)	0 (0.0)	70 (21.6)	145 (23.7)	0 (0.0)	597 (22.7)	21.1	24.3
	WC	79 (34.2)	381 (34.5)	65 (46.8)	76 (34.6)	1 (100.0)	130 (40.1)	210 (34.4)	0 (0.0)	942 (35.8)	34.0	37.6
	EC	38 (16.5)	70 (6.3)	6 (4.3)	15 (6.8)	0 (0.0)	64 (19.8)	39 (6.4)	0 (0.0)	232 (8.8)	7.8	10.0
	NW	0 (0.0)	3 (0.3)	1 (0.7)	1 (0.5)	0 (0.0)	2 (0.6)	3 (0.5)	0 (0.0)	10 (0.4)	0.2	0.7
	NC	0 (0.0)	2 (0.2)	0 (0.0)	0 (0.0)	0 (0.0)	0 (0.0)	0 (0.0)	0 (0.0)	2 (0.1)	0.0	0.3
	FS	1 (0.4)	2 (0.2)	0 (0.0)	0 (0.0)	0 (0.0)	0 (0.0)	4 (0.7)	0 (0.0)	7 (0.3)	0.1	0.6
	MP	0 (0.0)	9 (0.8)	1 (0.7)	2 (0.9)	0 (0.0)	0 (0.0)	6 (1.0)	0 (0.0)	18 (0.7)	0.4	1.1
	LP	5 (2.2)	24 (2.2)	0 (0.0)	6 (2.7)	0 (0.0)	1 (0.3)	24 (3.9)	0 (0.0)	60 (2.3)	1.8	2.9
	TOTAL	231 (8.8)	1106 (42.0)	139 (5.3)	220 (8.4)	1 (0.0)	324 (12.3)	611 (23.2)	2 (0.1)	2634 (100.0)		
95% confidence interval (Score (Wilson) method)	Lower CL	7.8	40.1	4.5	7.4	0.0	11.1	21.6	0.0			
	Upper CL	9.9	43.9	6.2	9.5	0.2	13.6	24.9	0.3			

These numbers include multiple occurrences on each website

*GP* Gauteng*, KZN* KwaZulu Natal*, WC* Western Cape *EC* Eastern Cape*, NW* North West*, NC* Northern Cape *FS* Free State *MP* Mpumalang*a LP* Limpopo

Based on the three measures of use, adjust(-ing/-ment), manipulate manipulate(-ion) and wellness were used in more webpages and were used more often and innate intelligence and vital(-ism/-istic) were very rarely used. There was some variation in ranking for alignment, holism(-tic) and subluxation depending on what measure was used. Subluxation was used in fewer webpages than alignment and holism(-tic) but when it was used it was used more often than the other two terms.

It was also of interest to examine the combination of terms used by webpages. Number of different terms used per webpage is shown in Table 4. The highest number of webpages cite two (*n* = 70;20.8%) or three (*n* = 68;20.2%) of the eight terms. No webpages cited all eight or seven of the eight terms. Sixty webpages (17.9%) used none of the eight terms.

**Table 4:** Prevalence of multiple term use among 336 South African chiropractic webpages

Number of different terms used	Number (n)	Percent (%)	95% confidence interval (Score (Wilson) method)	
			Lower CL	Upper CL
None	60	17.9	14.1	22.3
One of terms	55	16.4	12.8	20.7
Two of terms	70	20.8	16.8	25.5
Three of terms	68	20.2	16.3	24.9
Four of terms	58	17.3	13.6	21.7
Five of terms	19	5.7	3.7	8.7
Six of terms	6	1.8	0.8	3.8
Seven of terms	0	0.0	0.0	1.1
All eight terms	0	0.0	0.0	1.1

Bivariate analysis was used to establish if there was a significant linear relationship between number of times different terms were used. A significant positive linear relationship would demonstrate when use for one term was low or high use of another term would be similarly low or high. Significant positive correlations were observed (Table 5) for the following pairs of number of times used: adjust(-ing/-ment), with subluxation (Pearson r = 0.568); adjust(-ing/-ment), with alignment (Pearson r = 0.391); subluxation with alignment (Pearson r = 0.323); adjust(-ing/-ment), with manipulate(-ion) (Pearson r = 0.311); wellness and subluxation (Pearson r = 0.216); wellness and holism(-tic) (Pearson r = 0.211); and manipulate(-ion) and alignment (Pearson r = 0.158). All the aforementioned correlations were significant at the 0.001 level. Other pairing of terms, such as adjust(-ing/-ment), and wellness were not significantly correlated.

**Table 5:** Pearson r correlations between number of times different terms were used

	Manipulate(-ion)	Wellness	Alignment	Holism(-tic)	Subluxation
Adjust(-ing/ment)	0.311	0.047	0.391	− 0.004	0.568
Manipulate(-ion)		− 0.019	0.158	0.034	0.086
Wellness			0.112	0.211	0.216
Alignment				0.022	0.323
Holism(-tic)					0.019
Subluxation					

Significance levels are 0.001, 0.01, 0.05; strong correlation r = 0.50–1.00, moderate correlation r = 0.30–0.49, weak correlation r = 0.10–0.29 [43]

The bivariate analysis provides further evidence that some pairs of terms were more likely to be related than other pairs.

### Analysis of use of terms by sociodemographic variables

Number of webpages that cited a term at least once was used as the indicator to test if sociodemographic characteristics were associated with use of terms. For this analysis only the three most frequently reported provinces—Gauteng, KwaZulu-Natal and Western Cape—and the two most frequently reported universities—UJ and DUT—were included. Years in practice was recoded into categories 1–5 years, 6–10 years, 11–15 years, 16–20 years and 21 or more years. Only statistically significant results are reported. There were no significant associations between place of study and proportion of webpages using different terms.

Male chiropractors were more likely to use the term manipulate(-ion) on their webpages. Of the male chiropractors, 104 (61.5%) used manipulate(-ion) at least once on their webpage compared with 56 (47.1%) of female chiropractors (Pearson chisquare = 5.929, df = 1, *p* = 0.015). For other terms similar proportions of male and female chiropractors cited the term on their webpage.

Chiropractors with fewer than six years and those with over 20 years in practice were less and more likely to use the term alignment on their webpage respectively. While between 31.3% and 33.3% of chiropractors with 6–10 and 16–20 years of practice used the term alignment at least once on their webpage, just two (10.0%) of chiropractors with 1–5 years practice and ten (62.5%) with 21 years or more in practice featured the term on their webpage. This association was statistically significant (Pearson chiquare = 11.168, df = 4, *p* = 0.025).

Frequency of use of three terms show an association with province. Adjust(-ing/-ment), was more likely to be used by chiropractors in WC and Gauteng than those in KZN. Seventy-four (73.3%) and seventy-one (70.3%) of chiropractors in WC and Gauteng included at least one citation of adjust (-ing/-ment), on their webpages compared to just 49 (48.5%) of chiropractors in KZN. This association was statistically significant (Pearson chisquare = 16.020, df = 2, *p* = 0.001). Wellness was more likely to be used by chiropractors in the WC where 44 (43.6%) of chiropractors in WC included at least one instance of wellness on their webpage compared to 32 (31.7%) and 28 (27.7%) in Gauteng and KZN, respectively. This association was statistically significant (Pearson chisquare = 6.090, df = 2, *p* = 0.048). Holism(-tic) was more likely to be used by chiropractors in the WC where 41 (40.6%) of chiropractors in WC include at least one instance of holism(-tic) on their webpage compared to 28 (27.7%) and 19 (18.8%) in KZN and Gauteng, respectively. This association was statistically significant (Pearson chisquare = 11.755, df = 2, *p* = 0.003).

## Discussion

The words adjustment, manipulate and wellness were the three most commonly used profession-specific terms found on South African chiropractors’ webpages with 82.1% of those webpages using a chiropractic-related word at least once. This proportion is similar to that of Australian and United Kingdom (UK) chiropractor webpages which showed an 85% and 75% prevalence of chiropractic-specific words, respectively. This suggests that South African chiropractors may be similarly inclined to promote the more traditional unsubstantiated chiropractic historical beliefs [28, 30, 44, 45]. Interestingly, the words adjustment (64.3%) and wellness (12%) in this study were also found to be the most commonly used terms in both the Australian (77% and 33%, respectively) and UK (68% and 5%, respectively) samples suggesting that South African chiropractors may also feel the need to distinguish themselves from other manual therapists and professions by leveraging the word adjustment [28, 30, 44, 45]. This creates an unanticipated dilemma in that as the chiropractic profession tries to maintain its identity, it may be further isolating itself from the scientific community.

This preference within South African chiropractors of the word ‘adjustment’ rather than the word ‘manipulation’ may arise from the notion that an adjustment is more specific in its intention having broader mechanical and neurological effects that are perhaps absent in the type of manipulation provided by other health-care professionals [28, 46, 47]. However, the majority of published literature in general heath sciences peer-reviewed journals, classically recognizes the term "manipulation" rather than "adjustment" [48, 49], allowing for a potential miscommunication inter-professionally.

The moderate to strong correlational relationship between the prevalence of the words alignment, adjustment and wellness to the word subluxation found in the South African sample is somewhat predictable considering these terms are used to convey similar concepts. Early alignment-subluxation based chiropractors used radiographic imaging to study spinal alignment to identify the location of a ‘subluxated’ or misaligned vertebrae, a practice that is strongly discouraged in allopathic medicine and evidence-based chiropractic today [50]. Likely motivations for the alignment-subluxation based chiropractors are anecdotal or not being current in the evidence-based literature. Models that consider joint fixation/locking, intra-articular block, inter-articular adhesions, inter-discal block, muscle spasm, myofascial cycle, and periarticular fibrosis and adhesions [46] should alternatively be advocated by chiropractors. The weak correlational relationship observed, in this sample, between the words holism and wellness, holism and manipulation, subluxation and manipulation and the minor but tangible prevalence of these vitalistic constructs is evidence of a lack of empirical coherence within the chiropractic profession [28]. About two-thirds of South African chiropractors indicated that they practiced diversified chiropractic [27] which implies that a third practice other forms of chiropractic, vitalistic chiropractic being one possible form. The internal identity variances of the South African chiropractic profession, albeit in the minority, could only further confound the standing this complimentary profession has with conventional modern medicine which in turn is aggravated by the lack of the use of a common science-based language for effective interprofessional communication. Accurate and consistent communication between the health care professions benefits not only the professions themselves, but most importantly the patients [51].

Another relevant association was observed between the number of years a South African chiropractor was in practice to both the term ‘alignment’, where the longer the chiropractor was in practice (the older he/she is) the more likely they were to use chiropractic-related terminology (and those practicing for a very short time were much less likely to use the term than those practicing for a moderate time). It is possible that these results could be attributed to the adoption of the International Chiropractic Education Collaboration, Clinical and Professional Chiropractic Education Position Statement [29], post-2014. Most of the webpages (66.6%) in this sample represented chiropractor’s that received their chiropractic education and chiropractic degrees at a South African university. Both South African institutions conform to the International Chiropractic Education Collaboration, Clinical and Professional Chiropractic Education Position Statement that states that ﻿effective communication in a language that is clearly understood by all stakeholders in health-care should be used to better communicate between health-care teams. It also negates the teaching of the vertebral subluxation complex as a vitalistic construct and a cause of disease [29]. Thus, recently qualified, younger South African chiropractors, educated at South African institutions, use chiropractic-related terminology less because their educational institutions have upheld this position statement and provide evidence based teaching and learning.

This study showed that male South African chiropractors were more likely to use the word ‘manipulation’ on their webpage compared to their female counterparts. Most studies investigating chiropractic sex differences focus on the number of working hours and number of patients seen weekly. One study shows that female chiropractors spend more time with their patients on direct patient care. They also found that female chiropractors treated more patients with acute conditions, whereas male chiropractors treated more chronic conditions [52]. No studies currently exist that explore the use of chiropractic terminology between male and female chiropractors. However, as in many countries and in South Africa, the profession was primarily male dominated in terms of numbers of practitioners in the past and there would be a higher percentage of males in the older chiropractic population who were educated using these dated terms [52].

Chiropractors’ webpages in the Western Cape province showed the highest overall prevalence for the use of chiropractic-related terminology—specifically terms adjustment, wellness and holistic (but not the other terms)—when compared to the other two predominant provinces (KZN and Gauteng). The average age of chiropractors in the Western Cape Province is 37 years [53]. According to the list of the 72 chiropractors registered with the Chiropractic Association of South Africa (CASA) in the Western Cape, 53% are male. More noteworthy, the Western Cape exceeded the Gauteng province in chiropractic-related term prevalence, which is more densely populated with chiropractors, with Gauteng having 172 CASA registered chiropractors [54]. While, this study did not seek to determine differences between provinces or regions, the demographic of the majority of the chiropractors in the Western Cape may play a role in the increased prevalence of chiropractic-related terminology on their webpages.

### Strengths

This study advances on previous studies as it adds similar data based on a South African perspective while including gender and number of years in practice as variables to be considered, something not previously reported before.

### Limitations

Google search is not impartial to webpages that have not been skilfully marketed so this may have had an unaccountable effect on the results. The prevalence of chiropractic-related terms on a chiropractor’s website is not an automatic representation of their beliefs. The results found in this study are specific to the educational and professional contexts of chiropractic in South Africa that may not be directly generalised to other parts of the world. Chiropractors that work in group settings or do not have webpages remain unaccounted for. Counting words and determining the context of the words used on a website opens up the possibility to human error or oversight. This study did not investigate any differences found between provinces or regions.

## Conclusion

Two-thirds of South African chiropractors use at least two chiropractic-related words on their webpages with a partiality to the words adjustment, manipulate and wellness. While South African chiropractors seem attached to historical underpinnings of chiropractic to a lesser extent than Australian and UK chiropractors, chiropractic-related terminology does seem to be used by the chiropractors webpages in this study to distinguish their professional identity. The vitalistic chiropractic opinion, albeit in the minority, has survived to some extent. Evidence-based education has had a positive influence on the way chiropractors communicate and on the prevalence of chiropractic-related terminology. Certain demographics such as older age, the male sex and regional location in the Western Cape Province in South Africa seem to show increased usage of chiropractic-related words. Chiropractors in South Africa may have difficulty integrating and communicating effectively with other medical professionals in interdisciplinary settings as their interprofessional communication skills seem to be more informed by chiropractic philosophies rather than medical perspectives. Further investigations are warranted on the perceptions of chiropractic communication competency and interpretation by other health professionals.

## Supplementary Information

**Additional file 1.** References for Supplementary Material.

**Additional file 2.** Further descriptions of chiropractic-related terms.

## Data Availability

The datasets used and/or analysed during the current study are available from the corresponding author on reasonable request.
