# Supplementary material for: The prevalence of chiropractic-related terminology on South African chiropractors’ webpages: a cross-sectional study
Source: Chiropr Man Therap. 2023 Apr 3;31:11. doi: 10.1186/s12998-023-00483-3 (PMC10071643; doi:10.1186/s12998-023-00483-3)
Supplement: Supplementary file 1 — Additional file 1. References for Supplementary Material. [file 12998_2023_483_MOESM1_ESM.docx]

**References for Supplementary Material**

1. Vernon H. Historical overview and update on subluxation theories, Journal of Chiropractic Humanities. 2010; 17(1):22–32. doi:10.1016/j.echu.2010.07.001
2. Funk MF, Frisina-Deyo AJ, Mirtz TA, Perle SM. The prevalence of the term subluxation in chiropractic degree program curricula throughout the world, Chiropractic and Manual Therapies. BioMed Central Ltd. 2018; 26(1): 24-26. doi:10.1186/s12998-018-0191-1
3. Homola S. Real orthopaedic subluxations versus imaginary chiropractic subluxations. Focus on Alternative and Complementary Therapies. 2010 Dec 1;15(4):284–7. [doi.org/10.1111/j.2042-7166.2010.01053.x](https://doi.org/10.1111/j.2042-7166.2010.01053.x)
4. Simpson JK. The five eras of chiropractic & the future of chiropractic as seen through the eyes of a participant observer. Chiropractic and Manual Therapies. 2012 Dec; 20(1):1. doi:10.1186/2045-709x-20-1
5. Young KJ. Words matter: the prevalence of chiropractic-specific terminology on Australian chiropractors' websites. Chiropractic and Manual Therapies. 2020; 28(1):18. Published 2020 Apr 7. doi:10.1186/s12998-020-00306-9
6. Shekelle PG, Adams AH, Chassin MR, Hurwitz EL, Brook RH. Spinal manipulation for low-back pain. Annals of internal medicine. 1992; 117(7): 590-598. doi:10.7326/0003-4819-117-7-590
7. Paris SV. A History of Manipulative Therapy Through the Ages and Up to the Current Controversy in the United States. Journal of Manual and Manipulative Therapy. 2002; 8(2), 66–77. doi:10.1179/106698100790819555
8. Mintken PE, Derosa C, Little T, Smith. A Model for Standardizing Manipulation Terminology in Physical Therapy Practice. Journal of Orthopaedic & Sports Physical Therapy, 2008; 38(3), A1–A6. doi:10.2519/jospt.2008.0301
9. Pickar JG. Neurophysiological effects of spinal manipulation. The spine journal: official journal of the North American Spine Society, 2002; 2(5), 357–371. [doi.org/10.1016/s1529-9430(02)00400-x](https://doi.org/10.1016/s1529-9430(02)00400-x)
10. Gevers-Montoro C, Provencher B, Descarreaux M, Ortega de Mues A, & Piché M. Clinical Effectiveness and Efficacy of Chiropractic Spinal Manipulation for Spine Pain. Frontiers in pain research (Lausanne, Switzerland), 2021; 2, 765921. [doi.org/10.3389/fpain.2021.765921](https://doi.org/10.3389/fpain.2021.765921)
11. Haldeman S, Dagenals S, Budgell B, Grunnet-Nilsson N, Hooper PD, Meeker WC, Triano J. Principles and Practice of Chiropractic. 3rd ed. United States: McGraw-Hill Companies. 2005; pp. 74 & 370.
12. Jenkins HJ, Downie AS, Moore CS, French SD. Current evidence for spinal X-ray use in the chiropractic profession: A narrative review, Chiropractic and Manual Therapies. Chiropractic & Manual Therapies. 2018; 26(1):1–11. doi:10.1186/s12998-018-0217-8
13. Triano JJ, Budgell B, Bagnulo A, Roffey B, Bergmann T, Cooperstein R, Gleberzon B, Good C, Perron J, Tepe, R. Review of methods used by chiropractors to determine the site for applying manipulation. Chiropractic & Manual Therapies. 2013; 21(1), 36. doi:10.1186/2045-709x-21-36
