# Supplementary material for: The prevalence of chiropractic-related terminology on South African chiropractors’ webpages: a cross-sectional study
Source: Chiropr Man Therap. 2023 Apr 3;31:11. doi: 10.1186/s12998-023-00483-3 (PMC10071643; doi:10.1186/s12998-023-00483-3)
Supplement: Supplementary file 2 — Additional file 2. Further descriptions of chiropractic-related terms. [file 12998_2023_483_MOESM2_ESM.docx]

**Subluxation**

The term subluxation, although deeply rooted and central in the history of chiropractic,^[1]^ is of little clinical value today.^[2]^ This is emphasised by the fact that there is no consensus of its definition among the chiropractic profession in itself.^[3-4]^ Its main chiropractic concept was to describe the misalignment of a vertebra thereby applying pressure to surrounding nerves which cause eventual disease.^[1]^ This is far removed from the medical definition which is “a painful partial dislocation” that is grossly unstable due to ligamentous tearing, that has been recognised as that since the time of Hippocrates.^[3]^ Today, the teaching of the vertebral subluxation complex as a vitalistic construct that claims it is the cause of disease is unsupported by evidence and its inclusion in a modern chiropractic curriculum in anything other than an historical context, is inappropriate and unnecessary.^[2-3, 5]^ The fact that early chiropractors utilized the term ‘subluxation’ from medicine, but with a different definition, has caused decades of distrust and misconceptions of the chiropractic profession from medical colleagues.

**Adjust (-ing/-ment) and Manipulation**

Manipulation is a non-exclusive chiropractic word and is a commonly accepted medical term. Within the chiropractic community the word ‘adjustment’ is synonymous with manipulation.^[6]^ However, in mainstream medicine manipulation implies a passive movement that is skilfully applied to a joint or soft tissue that is of variable speed and amplitudes.^[7-8]^ The effects of an adjustment are largely mechanical, including an increase in joint mobility and a reduction of muscle spasm.^[19-10]^, There are a number of other elements that could be involved including joint fixation or locking, intra-articular block, inter-articular adhesions, inter-discal block, muscle spasm, myofascial cycle, and periarticular fibrosis and adhesions.^[11]^

**Alignment**

Chiropractic as a profession has a long history of biomechanical analysis of the spine through x-ray imaging. Early chiropractors used x-rays to study spinal alignment to identify the location of a ‘subluxated’ or misaligned vertebrae in order to correct the spinal alignment through the form of spinal manipulation.^[12]^ The measuring of alignment on x-rays has not been found to be a useful method to determine the location of spinal manipulation instead, radiographic imaging must only be used in accordance with the latest evidence-based guidelines.^[13^
